# Ewing Sarcoma and Ewing-Like Sarcoma and the Role of NKX2.2 Immunoreactivity

**DOI:** 10.7759/cureus.17391

**Published:** 2021-08-23

**Authors:** Asad Ullah, Margaret A Sinkler, Luis Velasquez Zarate, Alex Clavijo, Joseph White

**Affiliations:** 1 Pathology, Medical College of Georgia - Augusta University, Augusta, USA; 2 Orthopedic Surgery, Medical College of Georgia - Augusta University, Augusta, USA; 3 Pathology, University of Texas Medical Branch, Galveston, USA; 4 Pathology, Medical University of South Carolina, Charleston, USA

**Keywords:** ewing sarcoma, small round blue cell, immunostaining, ewing-like sarcoma, nkx2.2

## Abstract

Ewing sarcoma (ES) belongs to the family of “small round blue cell” tumors and its diagnosis currently involves a combination of immunostaining and molecular analysis. However, due to significant histological overlap with other tumors of the same family, accurate diagnosis has historically involved combining these results with clinical correlation. Recently, multiple studies have analyzed the role of NKX2.2 immunopositivity in the diagnosis of ES. NKX2.2, a downstream target of the Ewing sarcoma breakpoint region-Friend leukemia integration 1 (EWSR1-FLI1) fusion, has been identified as a potential stain to differentiate ES and Ewing-like sarcoma from other small round blue cell tumors. In this study, we examine the histopathological interpretation of five patients. Four cases showed fluorescent in situ hybridization (FISH)-identified EWSR1 rearrangement. In one case, rearrangements of EWSR1 or FUS could not be detected, and a diagnosis of Ewing-like sarcoma was rendered. NKX2.2 was immunopositive in all five cases. Based on this limited dataset, NKX2.2 immunopositivity can significantly support the diagnosis of ES and has the potential to support the diagnosis of fusion-undetected Ewing-like sarcoma in appropriate clinical and histologic settings.

## Introduction

Ewing sarcoma (ES) belongs to the family of “small round blue cell” tumors, which encapsulate a broad differential including carcinomas, sarcomas, melanomas, lymphoblastic lymphomas, and some pediatric tumors such as neuroblastomas and nephroblastoma. Historically, the specific diagnosis of small round cell tumors has been difficult as they are morphologically alike. But the importance of proper diagnosis is critical as many in this class are highly aggressive malignant tumors. The typical pathologic findings of tumors in this class include small round, hyperchromatic undifferentiated cells that have an increased nuclear-to-cytoplasmic ratio [1].

ES is a primitive neuroectodermal tumor. With an annual incidence of 250-400 in the United States, ES is the second most common sarcoma in children and young adults. The peak incidence of ES occurs within the second decade of life [2]. Anatomically, the most common sites are the upper thigh and buttock followed by the upper arm and shoulder. The tumor typically originates in bone unless it appears in patients older than 30, in whom it more commonly affects soft tissues. In long bones, the tumor commonly arises from diaphysis or metadiaphysis. ES classically presents with neurologic deficits as it tends to develop near major nerves. When ES is localized, the five-year survival rate is close to 70%, and hence valid modes of diagnosis are crucial. But when ES is metastatic, the five-year survival rate drops to 30%. The current treatment strategy for ES focuses on systemic treatment using chemotherapeutic agents. As of now, new modern therapies including monoclonal antibodies, small molecules, and immunotherapy have not shown much success in ongoing trials.

Many bone tumors are identified at initial presentation using basic radiographs. Lesions associated with ES are typically difficult to identify on radiographs and require more advanced imaging modalities such as CT and MRI. The radiographic margins of bone tumors play a role in determining the growth rate and behavior, which can predict if the lesion is likely benign or malignant. ES classically has a type III margin, meaning that it is permeative [3]. Additionally, the lesion characteristic for ES appears osteolytic with a multilayer periosteal reaction that is commonly referred to as “onion skin-like appearance” [1]. For ES, radiographic imaging is the current method for assessing tumor burden at diagnosis, monitoring response to therapy, and detecting disease recurrence.

Currently, the gold standard for the diagnosis is fluorescent in situ hybridization (FISH)-utilizing probes for the Ewing sarcoma breakpoint region (EWSR1) [4]. Classically, ES is characterized by reciprocal chromosomal translocations involving a member of the TET or FET family of genes with a member of the ETS family of transcription factors. The TET and FET families include the following genes: TLS, FUS, EWSR1, and TAF15. The most common translocation involved in ES is EWSR1 at 22q12 and Friend leukemia integration 1 (FLI1) at 11p24 creating the oncogenic transcription factor EWSR1-FLI1 in approximately 85% of cases [5]. Other common translocations include fusion with ERG (5-10%), ETV1, E1AF, and FEV (each <1%) [6].

Ewing like-sarcomas form part of a subset of tumors morphologically resembling ES with either non-EWSR1-ETS fusions or lacking known genetic abnormalities. These Ewing-like sarcomas histologically overlap with ES. The recent application of genomic methods has led to the discovery of new recurrent translocations in the pathologic spectrum of undifferentiated round cell sarcomas, including CIC-DUX4 fusion, resulting from either a t(4;19) or t(10;19) translocation, CIC-FOXO4 fusion, and BCOR-related fusions involving CCNB3, MAML3, and ZC3H7B gene partners [7,8]. In a recent study, researchers have reported that Ewing-like sarcoma (CIC-rearranged sarcoma) varied considerably from ES [9].

While using FISH techniques is the current gold standard in diagnosis, EWSR1 fusions are common in other mesenchymal neoplasms such as clear cell sarcoma, desmoplastic small round cell sarcoma, and extraskeletal myxoid chondrosarcoma and are not identified in Ewing-like sarcomas. Additionally, molecular detection of specific fusion genes is costly, laborious, and oftentimes of limited availability. Therefore, immunohistochemical (IHC) techniques are often used to aid in the diagnosis.

Recently, the use of NKX2.2 has emerged as a potential IHC target for the diagnosis of ES. NKX2.2, a homeodomain-containing transcription factor, is a downstream target of EWSR1-FLI1, which plays a role in neuroendocrine glial differentiation [10]. Early research has demonstrated NKX2.2 positivity in 80-93% of ES cases but only in the typical EWSR1-FLI1 morphology [11]. By using an immunostain for NKX2.2, we intend to further study the potential effectiveness of NKX2.2 in the diagnosis of both ES and Ewing-like sarcoma.

## Case presentation

Five patients were diagnosed with ES or Ewing-like sarcoma. Our patients' ages ranged was from three to 22 years; they included three males and two females. The tumor locations included the right ileum (2), right fibula, sacrum, and cervical spine spanning C3 to C7. The initial patient presentation included pain and/or neurologic dysfunction including arm weakness and urinary incontinence due to the mass effect of extraosseous soft tissue extension. Clinical correlation and initial histopathologic interpretation with H&E and CD99 immunostains were used to establish an initial diagnosis. Following initial diagnosis, FISH techniques were used to further establish the diagnosis. FISH identified EWSR1 rearrangement in four cases. In one case, no rearrangement of EWSR1 or FUS was identified, and a diagnosis of Ewing-like sarcoma was rendered (Table 1). All five cases were immunostained for NKX2.2 (clone 74-5A5) using an optimized protocol of biotin-free, polymer-based immunoperoxidase.

**Table 1:** Patient characteristics Patient ages ranged from 3-22 with three males and two females. Four patients tested positive for EWSR1 rearrangement; one tested negative for EWSR1 and FUS and was diagnosed with Ewing-like sarcoma. NKX2.2 was immunopositive for all five cases NED: no evidence of disease

Age/sex	Location	Common clinical features	EWSR1	Follow-up
3/F	Sacrum	Right thigh pain, limp	18% (<2.2%)	Undergoing chemotherapy and proton therapy
8/F	Right ilium	Right hip pain, limp, fever	6.3% (4.4%)	Lung metastasis at presentation. Alive with the disease
8/M	Spine C3-7	Neck pain, right arm weakness	15.9% (4.4%)	NED at 14 months after completion of chemotherapy
14/M	Right fibula	Right knee pain	Negative for EWSR1 and FUS	Rib and spine metastases 20 months after completion of chemotherapy
22/M	Right ilium	Right hip pain	21.2% (4.4%)	Undergoing chemotherapy

**Figure 1:**
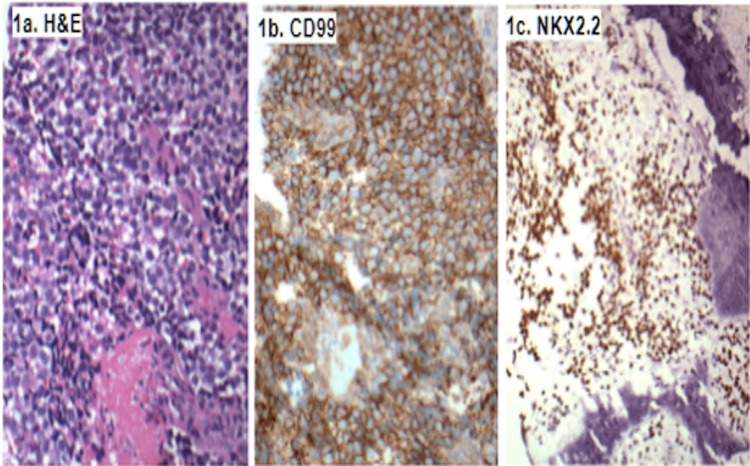
Immunostaining of samples H&E (1a) shows typical sheets of uniform small round blue cells with round/ovoid nuclei, distinct nuclear membrane, inconspicuous nucleoli, and an overall low mitotic rate. CD99 (1b) staining shows robust membranous immunoreactivity. Immunostaining for NKX2.2 (1c) shows nuclear immunopositivity in the same population of cells

All five cases showed typical ES architecture with sheets of uniform small round blue cells with generally oval nuclei, distinct nuclear membrane, inconspicuous nucleoli, and a low mitotic rate. Four cases showed FISH-identified EWSR1 rearrangement and were diagnosed with ES. In one case, rearrangements of EWSR1 or FUS could not be detected, and a diagnosis of Ewing-like sarcoma was rendered. The four cases of ES and a single case of Ewing-like sarcoma displayed diffuse NKX2.2 nuclear immunopositivity and CD99 membranous immunopositivity (Figure 1).

## Discussion

The family of small round blue cell tumors includes aggressive malignant tumors that are very difficult to distinguish from each other with our current diagnostic methods. Therefore, further development and understanding of unique cellular markers that can be used in the workup of such tumors is critical.

The diagnosis of ES relies on pathognomonic recurrent balanced translocations with rearrangement of EWSR1 gene on chromosome 22 in almost all cases with a few rare cases exhibiting FUS gene rearrangement that can be reliably identified using FISH. Even rarer are histologically overlapping Ewing-like sarcomas that lack these established gene rearrangements but may be positive for CIC-DUX4, BCOR-CCNB3 fusion, or other rare genetic abnormalities.

CD99 is an established IHC target used in the diagnosis of ES. CD99 is a surface glycoprotein encoded by the MIC2 gene found on the short arm of the X and Y chromosomes. CD99 typically shows a strong and diffuse membranous expression in a majority of ES cases. Other small round cell tumors such as anaplastic large cell lymphoma, lymphoblastic lymphoma and leukemia, poorly differentiated synovial sarcoma, rhabdomyosarcomas, and desmoplastic small round cell tumors can have mild, focal, or irregular CD99 expression [12]. The widespread use of CD99 in the diagnosis of ES is limited by its low specificity as it can be seen in the other tumors mentioned above.

Recent studies have identified additional IHC targets, such as NKX2.2, which may be useful for the diagnosis of ES and Ewing-like sarcoma. NKX2.2 is expressed in oligodendrocyte progenitors and has been thought to directly regulate oligogliogenesis [13]. In addition to the role in the development of the central nervous system, NKX2.2 plays a role in gastrointestinal and pancreatic endocrine cells. Recently, NKX2.2 has been shown to be involved in the oncogenesis of ES [14]. Staege compared the expression of NKX2.2 in neuroblastomas and ES and showed that it was differentially upregulated in ES [15]. All five of the patients included in our study showed positive immunoreactivity of NKX2.2.

The use of NKX2.2 is especially useful in the diagnosis of Ewing-like sarcoma considering that Ewing-like sarcoma lacks the translocation utilized in the FISH analysis. In a recent study from Harvard, researchers analyzed 40 genetically confirmed cases of ES for NKX2.2 expression, including four with atypical cytomorphology, four from unique locations (cutaneous and uterine), and three with confirmed EWSR1-ERG rearrangements [5]. NKX2.2 was positive in 37 (93%) ES cases, including atypical ES and tumors with known EWSR1-FLI1 or EWSR-ERG fusion. Focal NKX2.2 staining was noted in one poorly differentiated synovial sarcoma and three small cell carcinomas. The results from this study highlight the use of NKX2.2 in the diagnosis of Ewing-like sarcoma.

The future of the diagnosis of ES will likely involve the use of multiple IHC targets to increase the sensitivity and specificity of each individual test. A 2014 study in Japan investigated the combined use of CD99 and NKX2.2 and showed that NKX2.2 expression in CD99-positive non-ES tumors is a rarity, as only one out of three mesenchymal chondrosarcomas and one out of 15 small cell carcinomas were positive for NKX2.2 [10]. Therefore, the combined expression of NKX2.2 and CD99 appears to be much more specific for the diagnosis of ES than the expression of either marker alone. When used solely, CD99 had an overall specificity of 85% and overall accuracy of 88%. NKX2.2 had a sensitivity, specificity, and overall accuracy of 80%, 84%, and 82% respectively. When combined, specificity and overall accuracies were 98% and 92% respectively [10].

A recent IHC target includes paired box gene-8 protein (PAX8), which has not been studied widely. PAX8 is a nephric lineage transcription factor involved in the osteogenesis of the thyroid, kidney, and Mullerian system [12]. Currently, there are 12 documented cases of ES where the immunoreactivity of PAX8 has been investigated. The cases demonstrate immunoreactivity in 80% of the cases [12]. Further large-scale studies are needed to evaluate the role of this IHC target in ES and Ewing-like sarcoma diagnoses.

Additional methods of ES and Ewing-like sarcoma diagnosis include reverse transcriptase-polymerase chain reaction (RT-PCR) and next-generation sequencing (NGS). While these methods exist, they are often limited in use due to the cost and availability of technology.

The combination of the identification of non-EWSR1-ETS fusions including the CIC-rearranged sarcomas and BCOR-rearranged sarcomas and the use of NKX2.2 expression has the potential to reduce the number of unclassified round cell tumors. Additionally, the use of IHC to identify ES and Ewing-like sarcoma is a more financially feasible option for diagnosis compared to FISH techniques. This study provides further evidence for the utilization of the combination of NKX2.2 and CD99 in the diagnosis of ES and Ewing-like sarcoma.

## Conclusions

FISH techniques and IHC staining with CD99 have been used to aid in the diagnosis of ES; however, due to their low specificity, positive results have to be correlated with the full clinical picture to establish a diagnosis. NKX2.2, a downstream target of the EWSR1-FLI1 fusion, has been recently identified as a potential stain to differentiate ES from other small round blue cell tumors. Recent studies have further delineated ES and Ewing-like sarcoma regarding fusion status, presentation, and cellular morphology. Based on this limited dataset, NKX2.2 immunopositivity can significantly support the diagnosis of ES and potentially fusion-undetected Ewing-like sarcoma. Other less widely used diagnostic tools such as RT-PCR and NGS may not be available in all institutions. Therefore, we encourage our colleagues to use and rely on the combination of CD99 and NKX2.2 immunostains as these are cost- and time-effective diagnostic tools for small round blue cell tumors.
